# The Association between Short-term Exposure to Fine Particulate Matter and Outpatient Visit in Beijing, China

**Published:** 2017-11

**Authors:** Guangxi LI, Haitao LAN, Zhiguo LIU, Ting RUI, Jiapeng LU, Lingjie BIAN, Yinghui WANG, Shihan WANG, Hong ZHANG, Yongjun BIAN, Hui LI, Yuyan GUO, Shigang LIU, Liang LI

**Affiliations:** 1. Guang’anmen Hospital, Chinese Academy of Chinese Medical Sciences, Beijing, 100053, China; 2. Division of Pulmonary and Critical Care Medicine, Mayo Epidemiology and Translational Research in Intensive Care, Rochester, United States; 3. Fu Wai Hospital, Chinese National Center for Cardiovascular Diseases, Beijing, 100037, China; 4. Dept. of Oncology, Institute of Medicinal Biotechnology, Peking Union Medical College, Chinese Academy of Medical Sciences, Beijing, 100050, China

**Keywords:** Air pollution, Fine particulate matter (PM_2.5_), Association study, Short-term outpatient visits, Cardiopulmonary disease

## Abstract

**Background:**

We tried to investigate the effect of PM2.5 on daily counts of outpatient visits in the Guang’anmen Hospital to determine if short-term PM2.5 exposure with extremely high concentration affects cardiopulmonary function of Beijing residents.

**Methods:**

Outpatient visits and PM_2.5_ data from 01/11/2011 to 03/31/2013 were extracted from the Guang’anmen Hospital and the American Embassy in Beijing, respectively. Followed by using a semi-parametric generalized additive model (GAM) with time dependent covariates, we analyzed the association between PM_2.5_ concentrations and daily count of outpatient visits on Day 0, 1, 2, 3, 4 and 5 of PM_2.5_ exposure.

**Results:**

Overall, 284354 subjects were collected. There were significant associations of short-term PM_2.5_ exposures with outpatient visits for cardiopulmonary diseases (*P*<0.05). Specifically, a 10 μg/m^3^ increase in PM_2.5_ was positively associated with a 0.74% of increase in angina visit on the first day and 0.50% increased visit on the second day (*P*<0.05). With an increase in PM_2.5_, the cough and respiratory visits significantly decreased by 0.17% and 0.30% on the first day, respectively (*P*<0.05). However, there were significant positive associations of PM_2.5_ with increased cough and respiratory visits (increased by 0.17% and 0.10%, respectively) on the fifth day (*P*<0.05).

**Conclusion:**

Our association studies showed an instant effect of PM_2.5_ level on cardiovascular outpatient visit in the Guang’anmen Hospital in Beijing while a lag effect on respiratory outpatient visits.

## Introduction

Particulate matter plays an important role in air pollution, defined as the matter of aerodynamic equivalent diameter less than 10 μm (PM_10_). PM_2.5_, named as fine particulate matter no more than 2.5 μm in diameter, is major component of PM_10_ at 83%, drawn more attention recently in China (1). Carbonaceous materials, secondary inorganic aerosols (SIA), and mineral dust (Al, Ca, Fe, and Mg) are the three essential components of airborne PM in Beijing, as being main contributors to wintertime haze and adverse effects on people healthy (2). They are generated initially from natural and anthropogenic sources including biomass burning, vehicle exhaust, and industry, or secondary pollutants through heterogeneous chemical reactions (3). Air pollution in such circumstances in Beijing has turned more and more severe since 2000, due to rapid developments in Chinese economics, technology, and automotive industry, as well as population explosion in Beijing (4). Increased exposure to ambient particulate matter has a variety of adverse health effects including increased mortality, morbidity, hospital admission and outpatient visits (5–8). Researchers focused more on the adverse health effect of PM_2.5_ instead of PM_10_ because PM_2.5_ is a critical component in ambient particulate matter, and has been proved to be more significantly associated with acute health outcomes, as compared to PM_10_ (9). PM_2.5_ can remain suspended for longer time, easily go into indoor environment, and be transported over much longer distance (10). Several studies in toxicological and physiological fields had suggested that PM_2.5_ apparently affected human health, especially when sulfates, nitrates, acids, metals, and chemicals were adsorbed onto their surfaces (11–13). Those fine particles can go deeply and quickly into lower respiratory tract and even into alveolar region (14). Due to ‘spill-over effect’ of pulmonary inflammation or translocation into the circulation, PM_2.5_ might have adverse effect on respiratory and cardiovascular systems. There have been many scientific pieces of evidence showing the long-term effect of air pollution on respiratory and cardiovascular diseases (15–18). Less is known about the short-term influence of air quality on respiratory and cardiovascular diseases.

A few studies had discussed the relationship between PM_2.5_ short-term exposure and outpatient visit for respiratory and cardiovascular diseases. One study in Shanghai found that short-term PM_2.5_ exposure was associated with the increase of outpatient and emergency room visits (19). A survey in Beijing reported that the increase of PM_2.5_ results in elevation of respiratory mortality and morbidity. The growth rate of respiratory morbidity was highest even when PM_2.5_ was 40–60 μg/m^3^ (20). Although PM_2.5_ concentration was associated with respiratory and cardiovascular diseases, few of them showed the adverse health effect with such high PM_2.5_ levels in Beijing. In our study, the maximal concentration of PM_2.5_ was up to 491 μg/m^3^ with the average of 96.99 μg/m^3^. It is necessary to investigate the transient effect of PM_2.5_ with such extremely high concentration on human cardiopulmonary system in Beijing. It would help to detect adverse effect of extremely high level of PM_2.5_ after short-term exposure, as well as to understand further its physiopathological mechanisms of action of PM_2.5_.

**Fig. 1:**
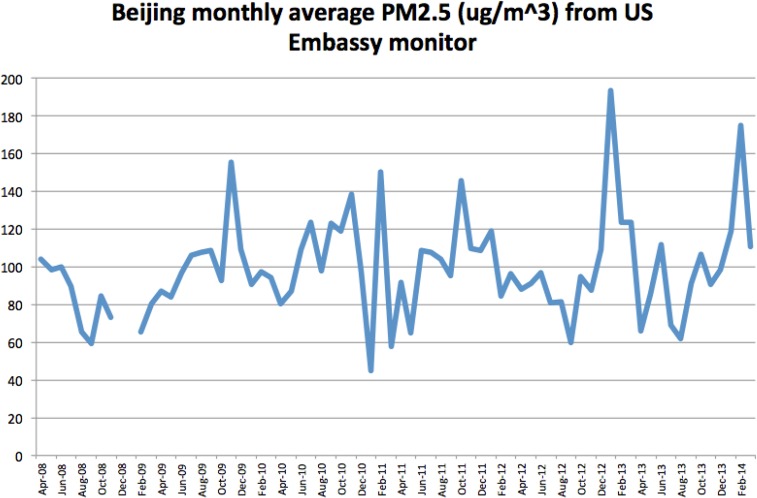
PM_2.5_ concentration trends

The composition and sources of PM_2.5_ particles vary dramatically by location, leading to different effects on public health. Beijing, as one of cities with the nation’s highest levels of PM_2.5_ concentration, traffic density, and residential population, is facing more and more severe problems that need public health studies to solve in this region. Fig. 1 showed Beijing’s monthly average PM_2.5_ levels from the data set containing nearly 50000 hourly readings from Apr 8, 2008, to Mar 31, 2014, according to the US Embassy monitor.

Especially in the recent decade in Beijing, air pollution mainly due to PM_2.5_ has gradually become one of the largest risk factors for both cardiovascular and respiratory diseases. As the out-patient medical treatment processes of Chinese medical care system are quite different from other countries, our patients usually, do not need to schedule appointments before they see their doctors in China. They could walk into hospitals directly and check in time to see available doctors once they feel uncomfortable. Moreover, the Guang’anmen Hospital is one of the largest academic hospitals in downtown of Beijing, in China and Guang’anmen Hospital in Beijing as shown in Fig. 2A and Fig. 2B.

**Fig. 2: A:**
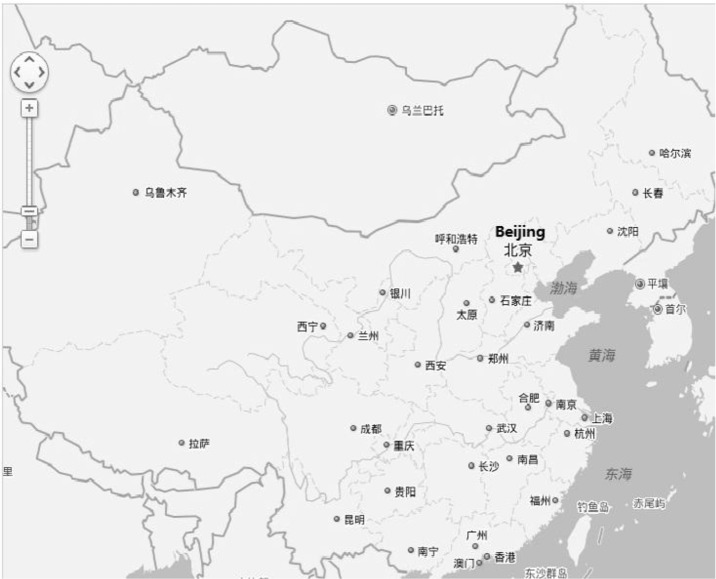
China map and the location of Beijing From Baidu Map V8.9.0

**Fig. 2: B:**
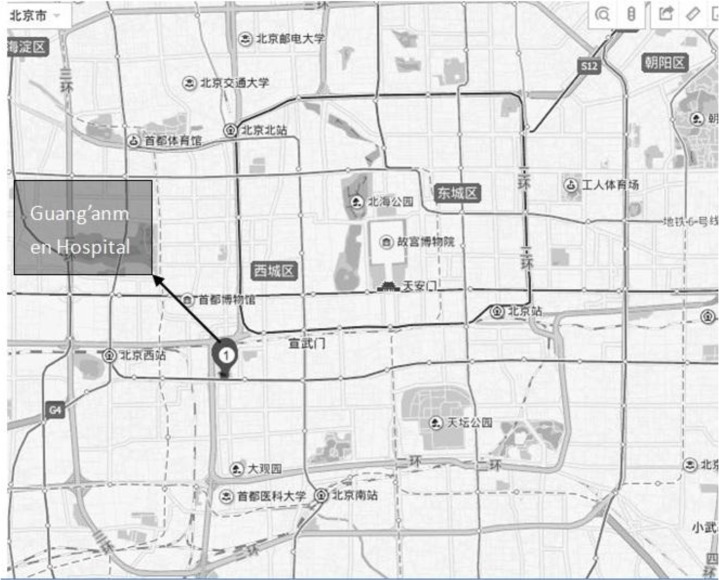
Beijing map and the location of Guang’anmen hospital^*^ From Baidu Map V8.9.0

Therefore, an outpatient visit for cardiopulmonary disease in the hospital would be a great indicator to reflect the short-term effect of air pollution in Beijing, differing from the PM_2.5_ long-term effect on hospitalization for cardiopulmonary disease.

In present study, we tried to investigate the effect of PM_2.5_ on daily counts of outpatient visits in the Guang’anmen Hospital to determine if short-term PM_2.5_ exposure with extremely high concentration based on time-series data from 2011 to 2013 in Beijing transiently affects cardiopulmonary function of Beijing residents.

## Methods

### Ethics statement

The study design has been approved by the IRB of the Guang’anmen Hospital. This was a retrospective study and no written informed consent was needed from the participants. Patients’ records/information was de-identified prior to analysis.

### Data collection

Beijing is located in the north of China with the world’s worst smog and about 21.7 millions of residential population in total. The daily PM_2.5_ concentrations from Nov 1^st^ in 2011 until Mar 31^st^ in 2013 were obtained from the American Embassy in Beijing. The data, including average daily temperature, dew point, relative humidity, air pressure, visibility and wind speed in Beijing, were collected from daily weather report by the Chinese Meteorological Administration.

The data of daily outpatient visits with 284354 patients were extracted from the electronic medical database of the Guang’anmen Hospital. The number of daily outpatient visits varied from 6500 to 11000, excluding the days when the out-patient clinic was closed. Only the visits for pulmonary and cardiovascular divisions were included in the study. In addition, the major complaints about outpatient visits were also extracted from the electronic medical records: a cough (ICD-10 R05xx01) and angina visits (ICD-10 I20.902).

### Statistical analysis

All the statistical analysis were performed using the software-Statistical Analysis System (SAS) for evaluating mean, minimum, median, maximum, standard deviation (SD) and interquartile range (IQR) values. Spearman correlation analysis was used to identify the association of temperature, dew point, relative humidity, air pressure, visibility, and wind speed, respectively. Since the generalized additive model (GAM) had been widely used for pollution studies (21,22), we also utilized time course of semi-parametric GAM to estimate the association of PM_2.5_ level with daily outpatient visit for either cardiovascular or respiratory disease, as well as other confounding factors including seasonality, long-term trend, meteorological factors and day of the week, respectively. We analyzed outpatient visits on Day 0, 1, 2, 3, 4 and 5 of PM_2.5_ exposure to display the daily effect of PM_2.5_ on risks in cardiovascular and pulmonary diseases. The relative risks (RRs) were calculated to determine the change of outpatient visit with a 10 μg/m^3^ increase in PM_2.5_.

## Results

Overall, 284354 patients had been collected during about one and half years in our study. The basic characteristics and daily meteorological factors, and PM_2.5_ concentrations during the same period were shown in details (Table 1).

**Table 1:** A descriptive statistics of meteorological factors, air pollutants and outpatient visits; Numbers shown are mean±SD or proportion

***Meteorological factors***			
Temperature (°)		8.35±11.84	
Dew point (°)		−1.35±13.78	
Relative humidity (%)		55.5±20.22	
Pressure (mpa)		1018.99±10.24	
Visibility (km)		9.74±7.7	
Wind speed (km/h)		9.95±5.53	
Atmospheric pollutants			
PM_2.5_ (μg/m^3^)		96.99±88.96	
Outpatient visits			
Respiratory division		Cardiovascular division	
Visits per day	210±104	Visits per day	340±159
Age (year)	51±11	Age (year)	61±9
Male (%)	47	Male (%)	52
Cough visits^*^	169±78	Angina visits^&^	10±9

* Cough visits: the visits whose major complaint was cough

& Angina visits: the visits whose major complaint was angina

The Spearman correlation analysis showed that the PM_2.5_ level in Beijing was significantly and positively correlated with dew point, humidity, while negatively associated with visibility and wind speed (*P*<0.05) (Table 2). PM_2.5_ exposure significantly affected the outpatient visit for angina as a chief complaint (RR=1.007, 95% confidence interval, CI: 1.003–1.012, *P*=0.003), while no statistically significant relationship between the PM_2.5_ level and daily cardiovascular division visit was found (RR=1.000, 95% CI: 0.999–1.001, *P*=0.742). PM_2.5_ level also affected the outpatient visits for both cough (RR=0.998, 95% CI: 0.997–0.999, *P*<0.001) and total respiratory outpatient visits (RR = 1.001, 95% CI: 1.000–1.002, *P*=0.013) (Table 3).

**Table 2:** The correlation coefficient of air pollutants PM_2.5_ and meteorological factors

	***Temperature (°C)***	***Dew point (°C)***	***Humidity (%)***	***Pressure (mpa)***	***Visibility (km)***	***Wind speed (km/h)***
PM_2.5_ (μg/m^3^)	0.095	0.323*	0.568*	−0.187	−0.752*	−0.547*

* *P*< 0.05, it can be thought of correlation analysis with statistical significance.

**Table 3:** The relative risk (RR) and 95% CI of PM_2.5_ (per 10μg/m^3^) on daily outpatient visits

	***Time***	***RR***	***95% CI***	***P value***
Cough	lag0	0.998	0.9971–0.9995	* P<0.001
	lag4	1.002	1.001–1.003	* P<0.001
Respiratory division	lag0	0.997	0.996–0.998	* P<0.001
	lag4	1.001	1.002–1.0018	* P=0.013
Angina	lag0	1.007	1.003–1.012	* P=0.003
	lag1	1.005	1.001–1.009	* P=0.021
Cardiovascular division	lag0	1	0.999–1.001	P=0.742
	lag1	1.002	1.001–1.002	*P<0.001

RR= relative risk, CI= confidence interval,

* P < 0.05, can be thought of correlation analysis with statistical significance.

lag0= current day=the first day, lag1= the second day, lag4= the fifth day

The results from the one-day lag effect analysis showed that PM_2.5_ exposure had statistically significant influences on both angina and total cardiovascular division visit right on the first day (*P*<0. 05) (Fig. 3A and 3B). No significant effect was observed on the other days. Interestingly, PM_2.5_ exposure had statistically significant effects on cough and total respiratory division visit on the fifth day, instead of the first day (*P*<0. 05) (Fig. 3C and 3D).

**Fig. 3: A:**
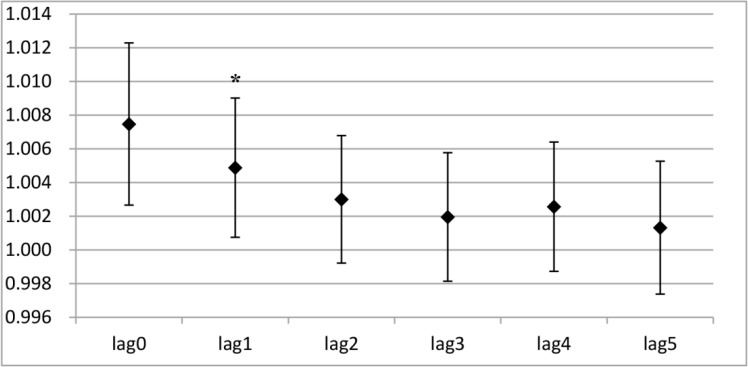
The RRs of PM_2.5_ in a single stranded effect on daily angina outpatient visits Y-axis indicates relative risk (RR) in the one-day lag effect analysis; * indicates *P*<0.05.

**Fig. 3: B:**
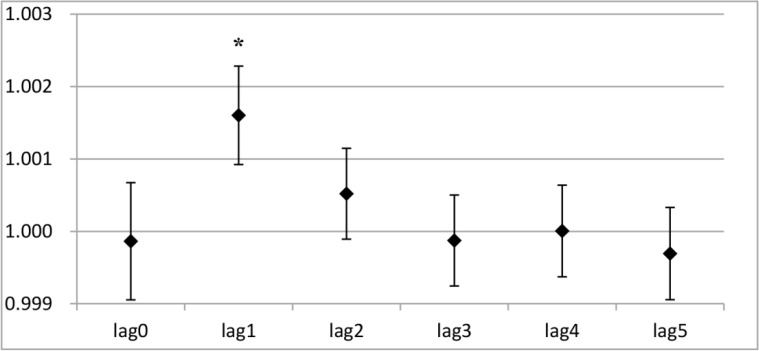
The RRs of PM_2.5_ in a single stranded effect on daily cardiovascular division outpatient visits Y-axis indicates relative risk (RR) in the one-day lag effect analysis; * indicates *P*<0.05.

**Fig. 3: C:**
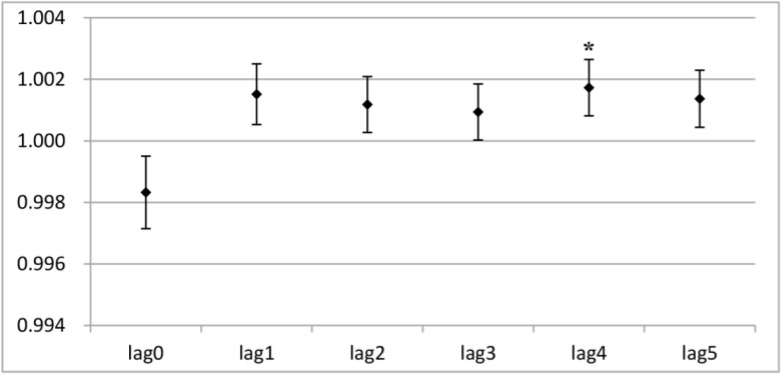
The RRs of PM_2.5_ in a single stranded effect on daily cough outpatient visits Y-axis indicates relative risk (RR) in the one-day lag effect analysis; * indicates *P*<0.05.

**Fig. 3: D:**
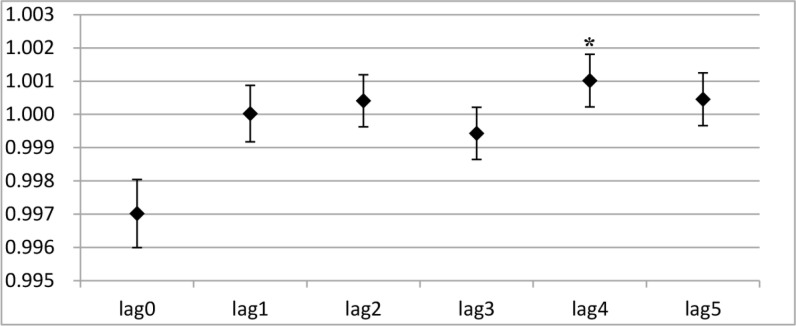
The RRs of PM_2.5_ in a single stranded effect on daily respiratory division outpatient visits Y-axis indicates relative risk (RR) in the one-day lag effect analysis; * indicates P<0.05.

After short-term exposure, a 10 μg/m^3^ increase in PM_2.5_ was positively associated with a 0.74% increase in angina visits on the first day, as well as a 0.50% increase on the second day (*P*<0.05) (Table 4). As shown in Table 4, with a 10 μg/m^3^ increase in short-term exposure to PM_2.5_ on the first day (lag0), the relative risk of outpatient visits for cough and total respiratory division significantly decreased by 0.17% and 0.30% (*P*<0.05). However, the cough and total respiratory division visits increased significantly by 0.17% and 0.10% (*P*<0.05) on the fifth day (lag4). No significant effect on the outpatient visits for cough and total respiratory diseases were found on the other days (*P*>0.05) (Table 4).

**Table 4:** Comparison of percentage increase (and 95% CI) in relative risk of outpatient visits associated with short-term PM_2.5_ exposure (per 10μg/m^3^)

		***Increased visits (%)***	***95%CI***	***P value***
Cough	lag0	−0.168	−0.286%–−0.049%	*P<0.05
	lag4	0.173	0.082%–0.264%	*P<0.05
Respiratory division	lag0	−0.299	−0.402%–−0.197%	*P<0.05
	lag4	0.101	0.022%–0.180%	*P<0.05
Angina	lag0	0.743	0. 265%–1. 221%	*P<0.05
	lag1	0.486	0.074%–0.897%	*P<0.05
Cardiovascular division	lag0	--	--	-
	lag1	0.16	0.092%–0.228%	P>0.05

## Discussion

The mechanisms underlying adverse effects of PM_2.5_ on respiratory and cardiovascular systems are still not fully understood. Pulmonary and systematic oxidative stress, inflammation, atherosclerosis, and related cardiovascular disease might be involved in. The increase in short-term air pollution exposure might provoke alveolar inflammation, with release of mediators capable of causing exacerbations of lung disease (23, 24).

Interestingly, we found that short-term PM_2.5_ exposure had a significantly instant effect on cardiovascular outpatient visits while a delayed effect on respiratory visits in Beijing. Our results showed increased angina visits on the first day, reflecting the acute effect (several hours to days) on cardiovascular response. The direct effect of air pollution might be attributed to the occurrence of rapid cardiovascular responses (within a few hours); such as increased heart rate and hypertension, leading to acute exacerbation of cardiovascular disease (25–27). One of the possibilities underlying mechanisms might be attributed to autonomic nervous system (ANS) dys-function, resulting in increased sympathetic activity and decreased parasympathetic activity, oxidative stress, and systemic inflammation. Reactive oxygen species (ROS) might also serve as acute potential mediators of PM_2.5_ effects on heart rate variability and other cardiovascular endpoints (28, 29). On the other hand, we observed the reduction of a cough and respiratory division visits on the first day and the increased visits on the fifth day. Similarly, a study in the United States showed that PM_2.5_ had lag effect on low respiratory infection (LRI) during the twenty-five months period (8). One of the explanations might be that patients usually restricted their outdoor activities including going to visit doctors on the foggy days, thus contributing to the decreased visits. Other unknown physiopathological pathways underlying mechanism of the delayed effect by short-term PM_2.5_ exposure might exist and needed to be further elucidated.

In addition, our study has the following limitations. Firstly, the nature of design for the retrospective and time series study limited our ability to draw a definite conclusion on causal effect between PM_2.5_ and outpatient visits. Secondly, we were unable to observe other types of air pollutants because the data were not available and these pollutants were not our major focus. Thirdly, the data for PM_2.5_ levels in Beijing were obtained from the monitoring station at the US Embassy in Beijing, because that was the PM_2.5_ assessment date only available for us during that time, data could reflect air quality of the entire city of Beijing. More accurate and adequate data for PM_2.5_ concentrations would be available for further analysis since Chinese government had set up more stations to monitor air quality in not only Beijing but also other surrounding areas. Fourthly, the present study was performed only in a single represented hospital in Beijing (the Guang’anmen Hospital) which might be not generalizable to all hospitals. However, we did investigate a long span of time with a larger number of visits, which reflected the effect of air pollution in Beijing on human health to a certain extent. Finally, the medical database for PM_10_ level in our hospital was limited. Due to this limitation, we did not investigate the relationship between PM_10_ and patient health alone, thus hard to discriminate from its complications from PM2.5. That would be considered in further analysis once we have both PM_10_ and PM_2.5_datasets available.

Furthermore, biomedical function studies on PM_2.5_ induced genomic instability, DNA damage, autonomic dysfunction, oxidative stress, inflammation and homeostasis would be required to elucidate molecular mechanisms by which PM_2.5_ might affect human cardiovascular and respiratory health. More direct or indirect evidence on individual-level data, including changes in genomics, epigenomics, proteomics and metabolomics caused by PM_2.5_, would be helpful to understand their physiopathological pathways and to prevent from those PM_2.5_-associated disease occurrences. We hence are planning to generate those data for further studies. Our findings would trigger the government to be aware of severe environment threatens and make corresponding healthy policies. That would also help to develop more effective biochemical tests and defensive medical devices.

## Conclusion

The short-term exposure to high level of PM_2.5_ pollution had an instant effect on cardiovascular outpatient visits whereas a lag effect on respiratory visits in Beijing. It might severely affect human cardiovascular and respiratory health, leading to a significant increase in outpatient visits for related diseases in Beijing.

## Ethical considerations

Ethical issues (Including plagiarism, informed consent, misconduct, data fabrication and/or falsification, double publication and/or submission, redundancy, etc.) have been completely observed by the authors.
